# Credentialing for eating disorder clinicians: a pathway for implementation of clinical practice standards

**DOI:** 10.1186/s40337-020-00332-1

**Published:** 2020-11-02

**Authors:** Siân A. McLean, Kim Hurst, Hilary Smith, Beth Shelton, Jeremy Freeman, Mandy Goldstein, Shane Jeffrey, Gabriella Heruc

**Affiliations:** 1 Australia & New Zealand Academy for Eating Disorders, Melbourne, Australia; 2 The Bouverie Centre, School of Psychology & Public Health, La Trobe University, Melbourne, 3056 Australia; 3 Eating Disorder Service, Robina Private Hospital, Robina, Australia; 4 School of Psychology, Griffith University, Gold Coast, Australia; 5 National Eating Disorders Collaboration, Melbourne, Australia; 6 Mandy Goldstein Psychology, Sydney, Australia; 7 Department of Psychology, Macquarie University, Sydney, Australia; 8 River Oak Health, Brisbane, Australia; 9 Royal Brisbane and Women’s Hospital, Brisbane, Australia; 10 School of Medicine, Western Sydney University, Campbelltown, Australia; 11 Eating Disorder Service, Northern Sydney Local Health District, Sydney, Australia

**Keywords:** Credentialing, Eating disorders, Treatment, Practice standards

## Abstract

Advances are needed to ensure safe and effective treatment is available for people with eating disorders. Recently developed clinical practice and training standards for mental health professionals and dietitians represent a significant step in this direction by providing a consensus statement on eating disorder treatment as a foundation on which to build competent practice. This commentary argues that a credentialing system could promote implementation of these practice standards through formal recognition of qualifications, knowledge, training and professional activities to meet minimum standards for delivery of safe and effective eating disorder treatment. Drivers for credentialing include the imperative to provide safe and effective care, promotion of workforce development in eating disorder practice and, importantly, readily available and transparent information for referrers, consumers, and carers to identify health professionals credentialed to provide eating disorder treatment. However, a number of factors must be considered to ensure that credentialing does not restrict access to care, such as prohibitively narrow criteria to become credentialed, absence of pathways for education, training, or professional development opportunities, and lack of consultation with or endorsement by stakeholders of the credentialing criteria, application and approval processes, and ways of identifying credentialed practitioners. Further work, including development of credentialing criteria and aligned training opportunities, currently being undertaken by the Australia & New Zealand Academy for Eating Disorders and the National Eating Disorders Collaboration in consultation with stakeholders in the eating disorders sector and health professions will advance understanding of the feasibility of a system of credentialing for eating disorders within Australia and New Zealand. The availability of clinical practice and training standards, supported by implementation pathways, including credentialing of eating disorders practitioners, aim to improve quality of life, reduce financial burden, and close the treatment gap.

It is well established that a treatment gap exists within eating disorders, whereby the majority of those in need of treatment do not receive appropriate care [1]. Individual level factors have been shown to obstruct treatment seeking, including fear of loss of control, failure to recognise the seriousness of symptoms, and fear of stigma [2–4]. Additionally, when consumers or carers do seek treatment, they often experience difficulty finding a suitably qualified and experienced treatment professional [5]. Systemic factors such as clinicians’ concerns about use of manualised evidence-based treatments and a shortage of clinicians trained in these can impede dissemination and implementation of evidence-based treatments so that even those who do seek care may not receive appropriate treatment [6]. These factors together can reduce patient and carer access to the type of treatment and care that is most likely to achieve optimal outcomes for those with eating disorders.

A significant step toward improving the likelihood that those in need will receive appropriate evidence-based care delivered by a health professional competent in eating disorder treatment was made with the recent development of the Australia & New Zealand Academy for Eating Disorders (ANZAED) clinical practice and training standards for mental health professionals and dietitians [7–9]. These standards provide a consensus statement on the complexity of treating eating disorders and formulate professional competencies required by mental health and dietetic professionals. However, further steps are required to ensure their dissemination across the sector and utilisation by health professionals. Among these steps, a credentialing system offers a potential pathway to support implementation by ensuring that participating clinicians have a minimum knowledge and level of training and professional development to provide safe and effective eating disorder treatment. This commentary paper considers the need for a credentialing system within the context of the Australian and New Zealand health systems and highlights important factors that might need to be considered within such a system for improving access to appropriate care that may in turn increase the likelihood of better patient outcomes. Acknowledging the differences among international health systems, it is anticipated that this commentary may serve as an example for the suitability of credentialing for other health jurisdictions.

Credentialing for eating disorders refers to a process of formal recognition of qualifications, knowledge, training, and development activities of professionals delivering eating disorder treatment. Although health professionals providing treatment for individuals with an eating disorder generally possess mental health, dietetic, and/or medical qualifications, and are typically recognised as appropriate to provide health care through registration with professional bodies, university training generally offers insufficient content on eating disorder management. It is widely acknowledged that due to the physical and psychological complexity of eating disorders, additional training and experience in their multidisciplinary treatment is needed to develop competence and confidence in their management. A credentialing system might formally recognise the level of training and experience that health professionals should attain to meet the minimum standards for the delivery of safe and effective eating disorder treatment.

Several incentives exist for credentialing in Australia and New Zealand. A principal driver is for people experiencing an eating disorder and carers to be able to access safe and effective care. Credentialing can achieve this in two main ways. By providing motivation and structure for the implementation of clinical practice standards, credentialing stands to contribute to the development of workforce capability and consistency of care for those with an eating disorder. Equally important, consumers and carers could exercise greater choice and control within a credentialing system in which they can be more informed about the clinicians who meet minimum standards for eating disorder treatment. Easy identification of credentialed eating disorder professionals across the system of care may particularly assist both primary care and consumers and carers. The primary care setting is often the gateway for accessing mental health treatment for eating disorders but knowledge about eating disorders, including referral sources, may be limited within those settings [5]. This may be reflected in the experiences of carers and consumers who have described the pathway to treatment as arduous [10]. A system of credentialing could offer a clear pathway for both referrers and consumers to find a credentialed health professional via a publicly available database.

Furthermore, practitioners may seek public recognition of their skills and experience through the credentialing system. This may be motivated by ethical or financial drivers or may also be viewed positively as a differentiator among early-career practitioners seeking to build their portfolio.

Key to the potential success of a credentialing system is ensuring that barriers do not deter use of the system or incur unwanted negative outcomes. First and foremost in these considerations is ensuring that enhancing the quality of treatment for eating disorders does not restrict access to care. Access would be negatively impacted if criteria to meet credentialing standards are narrow and reflective of “expert” levels of eating disorder knowledge, skills, and experience. Similarly, clinicians require access to adequate pathways for education, training, and continuing professional development to meet the minimum clinical practice standards for providing eating disorder treatment.

Also, to ensure clinician uptake, cost for credentialing must not be prohibitive and the process not too onerous so as to not exclude large numbers of professionals who provide effective treatment for eating disorders from participating. Other elements related to acceptability and uptake of credentialing by stakeholders also need to be considered. The system must be transparent, such that all of those affected by the implementation of credentialing (e.g. practitioners, consumers, carers, referrers, training providers, professional bodies) are consulted about key decisions to inform the credentialing system. Transparency must also be applied to the process by which credentialing applications are reviewed. In a related manner, the criteria against which applicants would be assessed for credentialing must be endorsed by professionals in the eating disorders sector to ensure the acceptability and utilisation of the system, as well as endorsed by consumers and carers who must be able to feel that they can trust the credentialing system if they are to engage with it as a means of accessing safe and effective treatment. Furthermore, the credentialing system must be fully accessible to referrers, consumers, and carers to ensure those in need can find an appropriate credentialed clinician in an efficient manner.

## Conclusion

In conclusion, we propose that a system of credentialing for eating disorder practitioners is needed in Australia and New Zealand. This system must balance the tension between access to treatment and quality of care. To advance the case for credentialing, work is being undertaken by ANZAED and the National Eating Disorders Collaboration to develop criteria for credentialing and to work with training providers to align training with these criteria. This work will be informed by the development of the ANZAED practice and training standards [7–9] and ongoing sector consultation that will account for the many roles within a multidisciplinary treatment team for eating disorders. Further work is also required to understand relevant local regulatory frameworks that might oversee the authority of an appropriate credentialing body. A credentialing system has the potential to contribute to workforce capability, recognition of and referral to credentialed health professionals, and limit delays in accessing evidence-based treatment for patients and carers alike. These advances stand to reduce the treatment gap, ultimately improving the process of accessing safe and effective care, to potentially enhance the likelihood of optimal treatment outcomes for people with eating disorders.

## Data Availability

Not applicable.
